# The Effect of a Soya-Based Dietary Fibre Beverage on Adiposity and Systemic Inflammatory Markers Among Overweight Adults: A Cluster-Randomized Controlled Trial

**DOI:** 10.3390/nu18121965

**Published:** 2026-06-18

**Authors:** Nurraihana Hamzah, Hamid Jan Jan Mohamed, Divya Vanoh, Wan Mohd Izani Wan Mohamed, Dzulkiflee Ismail, Majid Khan Majahar Ali, Nur Amanina Zainuddin, Siti Azhani Amran, Wan Rosli Wan Ishak

**Affiliations:** 1 School of Health Sciences, Universiti Sains Malaysia, Health Campus, Kubang Kerian 16150, Kelantan, Malaysia; raihana.h@usm.my (N.H.); hamidjan@usm.my (H.J.J.M.); divyavanoh@usm.my (D.V.); dzulkiflee@usm.my (D.I.); asitiazhani@gmail.com (S.A.A.); 2 School of Medical Sciences, Universiti Sains Malaysia, Health Campus, Kubang Kerian 16150, Kelantan, Malaysia; izani@usm.my; 3 School of Mathematical Sciences, Universiti Sains Malaysia, Minden 11800, Pulau Pinang, Malaysia; majidkhanmajaharali@usm.my; 4 Nutrition and Dietetics Unit, Hospital Pakar Universiti Sains Malaysia, Kubang Kerian 16150, Kelantan, Malaysia; nuramanina2796@usm.my

**Keywords:** soy dietary fibre, overweight adults, parallel intervention, weight loss, inflammation markers

## Abstract

**Background:** The increasing prevalence of overweight and obesity highlights the need for practical and sustainable dietary strategies for weight management. Although dietary fibre intake is associated with improved satiety and metabolic health, achieving recommended intake levels through whole foods alone remains challenging. Evidence supporting convenient, ready-to-consume fibre beverages in free-living overweight adults is also limited. Therefore, this study evaluated the effects of a soya-based dietary fibre beverage (SBB) on body composition and metabolic parameters in overweight adults. **Methods**: A 12-week parallel, cluster-randomized controlled trial was conducted on overweight university students and staff. An intervention group (IG) (*n* = 21) consumed the soya-based dietary fibre twice daily for 12 weeks, while the control group (CG) (*n* = 21) continued their habitual diet. **Results**: Significant group × time interactions were observed for body weight (*p* < 0.001), BMI (*p* = 0.021), waist circumference (*p* = 0.046), waist-to-hip ratio (*p* = 0.042), and body fat percentage (*p* = 0.004). The IG showed reductions in body weight (−1.12 kg), waist circumference (−4.29 cm), and body fat percentage (−0.73%), whereas the CG demonstrated minimal changes. No significant changes were observed in fasting glucose, lipid profile, CRP, or IL-6, suggesting no clinically significant adverse biochemical changes during the intervention period and supporting its short-term tolerability. Dietary analysis confirmed a marked increase in fibre intake in the IG (~50 g/day), indicating good adherence to the intervention. **Conclusions:** SBB supplementation improved body composition and central adiposity without affecting systemic inflammatory biomarkers and may represent a practical dietary approach for weight management in free-living overweight adults. Further studies are needed to confirm its long-term efficacy and safety.

## 1. Introduction

The rising trend in lifestyle-related health conditions has become a major public health challenge linked to dietary changes, urbanization, and sedentary lifestyles. Among these conditions, the increasing prevalence of overweight and obesity has drawn particular concern due to its strong association with non-communicable diseases, including cardiovascular disorders, type 2 diabetes, and metabolic syndrome. This trend has prompted a growing emphasis on identifying nutritional strategies that are not only effective but also practical and sustainable for long-term implementation in preventing and managing obesity. In this context, dietary fibre has attracted growing scientific interest due to its recognised roles in regulating appetite, glycaemic responses, lipid metabolism, and inflammatory activity. Numerous studies have reported that higher dietary fibre intake is associated with lower body weight, reduced adiposity, and improved cardiometabolic outcomes. Consequently, fibre-rich dietary components have emerged as promising and sustainable strategies for reducing obesity-associated health risks in diverse populations. Dietary fibre has received growing attention due to its capacity to influence appetite regulation, energy intake, glycaemic control, and lipid metabolism through multiple physiological mechanisms [1,2,3,4,5,6].

Previous research has shown that current approaches to increasing dietary fibre intake in overweight and obese groups have limited practicality and long-term sustainability. Traditional food-based strategies, such as consuming whole grains, fruits, and vegetables, are often challenged by factors like individual dietary preferences, limited food availability, time constraints during meal preparation, and gastrointestinal discomfort, all of which negatively impact long-term adherence [7,8,9,10,11,12]. Further research indicates that, due to these barriers, overall compliance with high-fibre diets remains consistently low among adults with overweight and obesity, despite clear guidance and proven health benefits [13,14,15,16]. To address these challenges, alternative methods, such as fibre-enriched beverages, have been examined, with some studies suggesting that liquid or semi-liquid formats might enhance acceptability and ease of consumption while minimally disrupting habitual eating behaviour [17,18,19,20,21]. In summary, while traditional food-based strategies face ongoing adherence challenges, fibre beverages could serve as a practical supplementary approach, highlighting the importance of systematically assessing their effectiveness in improving dietary fibre intake and supporting obesity management.

Research in this area commonly shows that increasing dietary fibre consumption improves both anthropometric and metabolic outcomes in overweight and obese people. Triffoni-Melo et al. [22] showed that higher fibre consumption is associated with reductions in body weight and body mass index, along with improved satiety and metabolic parameters. Findings from Liu et al. [23] indicate that both soluble and insoluble fibres contribute to metabolic improvements. However, soluble dietary fibre shows greater weight-loss and hypolipidemic potential than insoluble dietary fibre, potentially due to its viscosity and fermentability, which may enhance metabolic regulation [24,25,26]. Existing studies demonstrate that fibre-based interventions improve body weight regulation and metabolic health, thereby supporting further investigation of fibre-enriched products, including dietary fibre beverages, as a potential alternative strategy for weight management.

Dietary fibre plays a critical role in appetite regulation and satiety through physiological mechanisms that influence hormonal signaling and digestive processes. Dietary fibre intake was reported to enhance the secretion of satiety-related hormones, including glucagon-like peptide-1 (GLP-1) and peptide YY (PYY), while suppressing ghrelin, thereby contributing to reduced hunger and improved appetite control [6,27,28]. Besides that, certain fibres delay gastric emptying, prolonging feelings of fullness and subsequently reducing overall caloric intake [4]. Furthermore, studies on resistant starch indicate that its fermentation in the colon leads to the production of short-chain fatty acids, which are associated with metabolic and satiety-related benefits [29]. These findings demonstrate that dietary fibre influences appetite and energy intake through multiple, fibre-specific mechanisms; however, the limited integration of these effects within a single intervention, particularly in the form of fibre-rich beverages, highlights a gap in understanding their combined impact on both subjective appetite responses and objective metabolic outcomes in overweight populations.

Although fibre-enriched products are proposed as practical strategies to improve dietary adherence, the health effects of dietary fibre beverages have not been comprehensively evaluated across multiple physiological outcomes within a single intervention, especially among overweight adults. Addressing this gap, the present study aimed to evaluate the effects of a soya-based dietary fibre beverage on body weight and adiposity among overweight adults, which were the primary outcomes of interest. Secondary outcomes included appetite regulation, anthropometric measurements, biochemical parameters, and inflammatory biomarkers. By integrating anthropometric assessments, blood-based biomarkers, subjective satiety measures, and dietary intake evaluation, this study adopted a multidimensional approach to examining the efficacy of dietary fibre supplementation delivered through a functional beverage. Based on the established physiological roles of dietary fibre in promoting satiety, delaying gastric emptying, and modulating glucose and lipid metabolism, it was hypothesised that regular consumption of the soya-based dietary fibre beverage would enhance satiety, reduce body weight and adiposity indices, and favourably influence glycaemic, lipid, and inflammatory biomarkers compared with the control group. The use of a functional beverage as the delivery vehicle was intended to enhance practicality and adherence, thereby addressing key limitations identified in previous dietary fibre intervention studies.

## 2. Materials and Methods

### 2.1. Study Design and Setting

This study was conducted as a parallel, non-blinded, cluster-randomized controlled trial at the Health Campus, Universiti Sains Malaysia (USM), Kelantan, Malaysia, over 12 weeks, evaluating the effect of consuming a soya-based beverage (SBB) before breakfast and teatime in overweight adults. The study included two parallel groups (intervention and control). Ethics was approved by the Human Research Ethics Committee (HREC), Universiti Sains Malaysia (JEPeM Code: USM/JEPeM/KK/24111003). Clinical trial registration: The study was initially conducted under confidentiality constraints due to the involvement of an industrial partner (MELILEA (M) SDN BHD). For this reason, certain aspects of the project were subject to confidentiality agreements during the development phase. Following the removal of these restrictions, the clinical trial was retrospectively registered with ClinicalTrials.gov on 21 May 2026 (Registration No. NCT07614178), after completion of the study.

### 2.2. Sample Size

The sample size was calculated based on the primary outcome of body weight change, one of the pre-specified primary outcomes of the study, together with adiposity measures. The calculation was performed using the weight reduction reported by Nederveen et al. [30], assuming a significance level of 0.05 and a statistical power of 90% (β = 0.10). A 30% attrition rate was incorporated into the calculation. Using G*Power software (ver. 3.1.9.4; Heinrich-Heine-Universität Düsseldorf, Düsseldorf, Germany), the required sample size was determined to be 21 participants per group.

### 2.3. Study Population and Sampling

**Figure 1:**
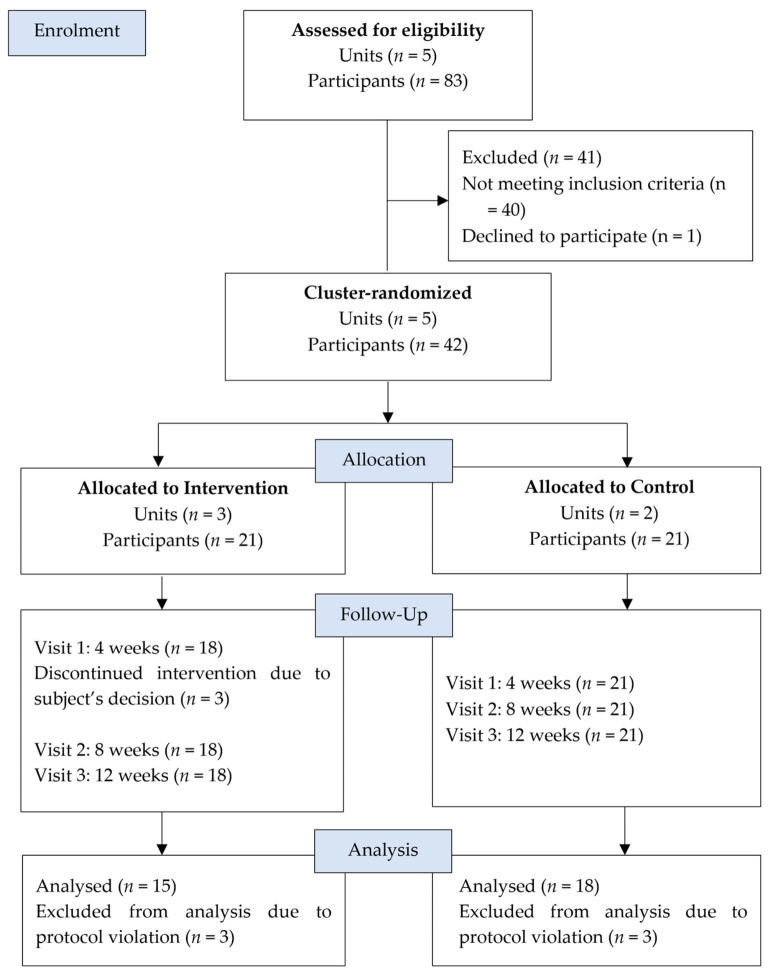
Consolidated Standards of Reporting Trials (CONSORT) 2025 flow diagram for subject inclusion in the intervention study.

The study population consisted of overweight students and staff working at the Health Campus, USM, Kelantan, Malaysia. A sample of 42 students and staff who were overweight were recruited for the study after screening for eligibility criteria (Figure 1). 

Inclusion criteria included: (a) age 18–50 years; (b) BMI 23–30 kg/m^2^; (c) physically inactive; (d) not having any allergies to any of the known food ingredients, especially milk and soy; (e) not on lipid-lowering and oral hypoglycemic drugs; and (f) no kidney diseases. The BMI range was selected based on the World Health Organization (WHO) recommendations for Asian populations, which recognize that Asians experience an increased risk of cardiometabolic diseases at lower BMI levels compared with Western populations. Accordingly, a BMI of ≥23.0 kg/m^2^ is considered overweight or at increased risk in Asian populations [31]. Participants were also required to be generally healthy and willing to comply with all study procedures throughout the intervention period.

Exclusion criteria were (a) pregnant or lactating mothers; (b) having known chronic disease conditions; (c) having a known allergy; (d) history for soy and milk or any of the ingredients in the formula; (e) history of any minor or major surgical procedure in the past 6 months; (f) currently on diet prescriptions or participating in regular physical activity sessions; (g) on weight loss drugs or consuming dietary supplements such as protein shakes, whey protein, casein, fruit juices or fibre-based supplements. Participants satisfying the eligibility criteria were enrolled according to their institutional affiliation. To minimise contamination between participants, allocation was performed at the cluster level rather than the individual level. The clusters were defined as institutional units within the Health Campus.

The institutional unit was used as the unit of randomization. Five institutional units within the Health Campus, Universiti Sains Malaysia (Hospital Pakar Universiti Sains Malaysia (HPUSM), School of Health Sciences (PPSK), School of Medical Sciences (PPSP), School of Dental Sciences (PPSG), and the Administration of Health Campus (Admin)) were defined as clusters. Prior to participant recruitment, cluster allocation was performed using Randomizer.org by assigning the five clusters to either the intervention or control arm. HPUSM, PPSP, and PPSG were allocated to the intervention arm, whereas PPSK and Admin were allocated to the control arm. Following cluster allocation, eligible participants were recruited from all five institutional units. All participants within the same cluster received the same study condition to minimize contamination between intervention and control participants. Because cluster allocation was completed before recruitment and the intervention involved consumption of a dietary fibre beverage, participant and investigator blinding was not feasible. Allocation concealment was not applied at the participant level because recruitment occurred after cluster assignment.

### 2.4. Recruitment

Students and staff from five institutional units within the Health Campus, Universiti Sains Malaysia (USM), Kelantan, Malaysia, were recruited voluntarily for this study through the distribution of research information posters via email and WhatsApp. The five institutional units had been previously randomized as clusters into either the intervention or control arm prior to participant recruitment. Initial screening was conducted to assess eligibility, including BMI measurements, medical history evaluation, and dietary intake screening. Eligible individuals were enrolled based on their respective institutional cluster assignment. A briefing session was conducted separately within each institutional unit to explain the study protocol and all relevant information in the consent form before written informed consent was obtained.

### 2.5. Interventions

The test group was provided with the soy-based dietary fibre beverage (SBB). The SBB was provided by the product owner (MELILEA (M) SDN BHD). However, the manufacturer/sponsor organization was not involved in any aspects related to the conduct of the study, including patient recruitment, follow-up, and analysis of data. The SBB (one serving = 53 g) contained 8.6 g of protein, 3.7 g of fat, 36.5 g of carbohydrate, 23.2 g of dietary fibre, 8.20 g of sugar, and 229.5 kcal.

The SBB was in powdered form, and participants were instructed to prepare the liquid shake by mixing the powder with 250 mL of cold water in a shaker that had been provided. They were asked to consume two servings of the beverage at least 30 min before each breakfast and teatime every day for 12 weeks. The intervention group was provided with a sufficient quantity of SBB sachets to last throughout the study period, with a label indicating the ingredients and the preparation instructions. The breakfast, lunch, and dinner meals were recommended as normal meals (the meals that they habitually eat). The intervention involved the consumption of the SBB powder while allowing participants to maintain their usual meal portions. There was no strict portion control, and participants could continue their habitual eating patterns. The focus was on incorporating the SBB as part of their daily diet without restricting overall food intake.

The control group was asked to continue their usual breakfast, lunch, and dinner meals during the study period. For both the intervention and control groups, participants attended a consultation regarding food intake following the Malaysian Food Pyramid Guideline and the Malaysian Healthy Plate (MHP), which emphasizes the Quarter Quarter Half (QQH) concept. Participants were advised to maintain their usual dietary habits and physical activity levels throughout the study. During the study period, the participants were not allowed to consume any other dietary supplements or participate in other weight loss programs.

Participation in this study requires fasting for 8–12 h before attending the baseline meeting (week 0) for fasting blood sample collection. A total of 10–12 mL of blood was drawn from a vein. Anthropometric measurements, including body weight, height, waist and hip circumference, and body fat percentage, were recorded. Participants in the intervention group received the study product, SBB powder, after completing the baseline session, with instructions on usage and daily dosage provided by the researcher. The control group maintained their usual dietary routine.

Anthropometric measurements, such as body weight, height, waist and hip circumference, and body fat percentage, were recorded on follow-up visits at weeks 4 and 8. The unused SBB powder was collected by the researcher, and a new supply was provided. The final session in week 12 followed the same procedure as the baseline session. Fasting for 8–12 h was required before blood sample collection, with 10–12 mL of blood drawn from a vein. Anthropometric measurements, including body weight, height, waist and hip circumference, and body fat percentage, were recorded. Any remaining SBB drink powder was returned to the researcher.

### 2.6. Measurement Tools

#### 2.6.1. Anthropometric Measurements

Anthropometric measurements were performed using calibrated equipment. Height was measured to the nearest 0.1 cm, as the maximum distance from the heels to the uppermost position on the head, with the individual standing barefoot and in full inspiration, using a calibrated stadiometer (Seca 769 portable stadiometer). Body weight was measured to the nearest 0.1 kg using a calibrated digital scale (Seca 769, Hamburg, Germany), with participants wearing indoor light clothing and emptying their bladders. Waist circumference was measured midway between the iliac crest and the lower rib margin at the end of normal expiration using a flexible plastic tape to the nearest 0.1 cm. Similarly, the hip circumference was measured at the widest level over the greater trochanter level to the nearest 0.1 cm. BMI was calculated as body mass in kilograms divided by height in meters squared (kg/m^2^), and the waist-to-hip ratio was calculated by dividing the measured waist circumference by the hip circumference.

#### 2.6.2. Body Composition

Body composition measurements were carried out to assess body fat mass, percentage of body fat, lean body mass, and body water. A calibrated body composition analyzer (TANITA SC-330, Tokyo, Japan) was used for this test.

#### 2.6.3. Dietary Records and Appetite

Dietary intake was assessed using a 3-day dietary record (two weekdays and one weekend day) at baseline, Week 4, Week 8, and Week 12 to capture habitual consumption patterns. Participants recorded all foods and beverages consumed, including portion sizes and preparation methods. Records were reviewed for completeness prior to analysis. Dietary data were entered into Nutritionist Pro™ Diet Analysis software version 7.8.0 (Axxya Systems, versin 2020, Redmond, WA, USA) to estimate mean daily energy and nutrient intake, including macronutrients and dietary fibre. For food items that are not available in the Malaysian Nutrient Composition of Foods, the USDA nutrient database was referred to. For mixed dishes that were not available in the database, local recipes were entered into the software.

Subjective appetite sensations were assessed using an eleven-point numerical rating scale (NRS), with anchors at 0 (“not at all”) and 10 (“very much”), to evaluate hunger, fullness, desire to eat, thirst, and prospective food consumption. Participants completed the questionnaire before and after breakfast, lunch, and dinner at each study time point (pre, week 4, and post). For analysis and comparability with conventional visual analogue scale (VAS) methods, NRS scores were linearly transformed to a 0–100 scale by multiplying each value by 10. Changes in appetite ratings (ΔVAS) were calculated as the difference between post-meal and pre-meal values for each meal (ΔVAS = post-meal − pre-meal).

#### 2.6.4. Biochemical Analysis

A total of 12 mL of venous blood was drawn from each participant by a trained phlebotomist following an overnight fast (8–10 h). The blood samples were distributed into four appropriately labelled tubes according to assay requirements (e.g., EDTA, serum separator, and fluoride oxalate tubes). Three tubes were immediately transported under controlled conditions to B.P Clinical Laboratory, Kota Bharu, Kelantan, for standardized biochemical analyses. These included fasting blood glucose, glycated hemoglobin (HbA1c), lipid profile, renal function tests, liver function tests, and C-reactive protein (CRP). All analyses were conducted using automated clinical chemistry analyzers following laboratory standard operating procedures. The remaining serum sample was processed on-site. Blood was allowed to clot at room temperature and centrifuged at 3000 rpm for 10 min to separate serum. The serum was aliquoted and stored at −80 °C until analysis. Interleukin-6 (IL-6) concentrations were quantified using a commercially available enzyme-linked immunosorbent assay (ELISA) kit (BioSwamp, Wuhan, China), according to the manufacturer’s instructions. All samples were analyzed in duplicate to ensure assay reliability.

### 2.7. Statistical Analysis

All analyses were performed using IBM SPSS Statistics version 29.0 (IBM Corp., Armonk, NY, USA). Statistical significance was set at *p* < 0.05. Continuous variables are presented as mean ± standard deviation (SD). For intergroup comparisons, independent samples t-tests for normally distributed variables and Mann–Whitney U tests for non-normally distributed variables were used. Repeated measurements were analyzed using linear mixed-effects models with restricted maximum likelihood estimation. Fixed effects included group (intervention vs. control), time (baseline, week 4, week 8, post-intervention), and group × time interaction. Participant ID was included as a random intercept to account for within-subject correlations. The covariance structure was selected based on the Akaike Information Criterion, with a first-order autoregressive structure yielding the optimal model fit. Analyses were conducted using available cases. Participants with missing outcome data due to dropout were excluded from the analysis dataset. No imputation was performed for missing data. IL-6 and C-reactive protein concentrations were log-transformed prior to analysis to satisfy normality assumptions. No additional covariates were included in the primary models.

## 3. Results

### 3.1. Participant Characteristics

Forty-two participants (9 males and 33 females), with a mean age (±SD) of 37.07 ± 7.13 years, were recruited from the randomized clusters and allocated according to cluster assignment. Three participants from the intervention group withdrew before the 12-week assessment due to personal reasons, including inability to tolerate taste and gastrointestinal discomfort. Consequently, 39 participants completed the follow-up assessment. No other clinically significant adverse events were reported among participants who completed the study. Among these, six participants were excluded from the final analysis due to protocol violations (IG = 3, CG = 3), resulting in a final analytical sample of 33 participants. Data from 33 participants (79% of those enrolled) were included in the final analysis. A flowchart illustrating participant recruitment, follow-up, withdrawals, exclusions, and final analysis is presented in Figure 1. The baseline characteristics of all participants including anthropometric, biochemical, body composition, and inflammatory parameters, are shown in Table 1. No significant differences were observed in any measured variable between the IG and CG at baseline (*p* > 0.05), confirming comparability between groups prior to intervention.

### 3.2. Effects on Clinical and Anthropometric

Table 2 shows the changes in anthropometric outcomes over the 12-week intervention period. SBB supplementation for 12 weeks results in statistically significant reductions in weight (*p* = 0.003), BMI (*p* = 0.02), and waist circumference (WC) (*p* = 0.27) compared to CG. The linear mixed model analysis revealed significant group × time interaction for weight (*p* < 0.001), BMI (*p* = 0.021), WC (*p* = 0.046), and waist-to-hip ratio (WHR) (*p* = 0.042). Hip circumference declined over time in both groups (time effect *p* < 0.001), but no significant interaction was observed.

Both the CG and the IG displayed relatively stable systolic and diastolic blood pressures across the 12-week duration, as no significant interactions were observed for systolic or diastolic blood pressure (Table 2).

### 3.3. Effects on Body Composition Parameters

Considering the changes in body composition (Table 3), the IG had a reduction of 0.73 ± 0.89 body fat percentage (BF%), whereas the CG gained 0.35 ± 0.67 BF% from baseline (*p* < 0.001), respectively. Similarly, fat mass in the IG showed a decline (−0.9 ± 1.10 kg), while the CG showed an increase in fat mass (0.38 ± 0.72) (*p* < 0.001), respectively. The linear mixed model analysis indicated significant group × time interactions in BF% (*p* = 0.004) and fat-free mass (FFM) (*p* = 0.047). However, no main effect of group or time was detected across all the measured body composition parameters.

### 3.4. Effects on Metabolic and Inflammatory Biomarkers

Changes in biochemical parameters are summarized in Table 4. A significant time effect was observed for fasting glucose (*p* < 0.001), with reductions in both groups; however, no significant group × time interaction was detected. Nevertheless, no statistically significant differences by group, time, or their interaction were observed for HbA1c and lipid profile parameters (total cholesterol, low-density lipoprotein cholesterol (LDL cholesterol), high-density lipoprotein cholesterol (HDL cholesterol), and triglycerides).

Over the span of 12 weeks, both the IG and the CG exhibited slight reductions in C-reactive protein (CRP); however, the changes were not statistically significant between groups. Interleukin-6 (IL-6) in both the IG and CG remained stable throughout the intervention. However, the linear mixed model analysis revealed no statistically significant differences regarding the group, time, or the interaction between group and time across all the measured inflammatory marker parameters.

### 3.5. Change in Diet

Although both groups maintained similar overall energy intakes, there were noticeable differences in specific macronutrient consumption (Table 5). Dietary assessment showed significant increases in fibre intake in the IG, rising from about 3 g/day at baseline to approximately 50 g/day during the intervention. Fibre intake in the control group remained low (3–6 g/day). Significant differences between groups were observed in dietary fibre, carbohydrate, and protein intake across time points. Carbohydrate intake increased over time in both IG and CG. Additionally, the IG showed greater increases in protein intake compared to the CG over time.

### 3.6. Effects of SBB on Subjective Appetite Sensations

Changes in subjective appetite sensations (ΔVAS) across the intervention period are presented in Table 6. No significant group × time interaction effects were observed for any appetite parameter, indicating that the temporal changes did not differ significantly between the control group (CG) and intervention group (IG). However, there were significant main effects of group observed for desire to eat snacks (*p* < 0.001), thirst (*p* = 0.019), and prospective food consumption (*p* = 0.007).

## 4. Discussion

This randomized controlled intervention demonstrates that a soya-based dietary fibre beverage (SBB) significantly improved anthropometric parameters and body composition in overweight adults over 12 weeks. The intervention group showed a slight reduction in weight, BMI, waist circumference, and body fat percentage, with functionally relevant changes in appetite regulation. Importantly, these effects occurred without adverse alterations in biochemical markers, supporting both the efficacy and safety profile of the intervention.

The most noticeable effect was observed in central adiposity. Waist circumference declined by over 4 cm in the intervention group, accompanied by a reduction in waist-to-hip ratio. Reductions in abdominal adiposity are clinically important, as visceral adiposity is strongly associated with adverse cardiometabolic outcomes [32,33,34]. Interestingly, waist circumference decreased to a greater extent than expected given the relatively small weight change (~1.1 kg), suggesting a targeted effect on central adiposity rather than overall body mass.

The body composition findings provide evidence of improved adiposity-related outcomes. Significant reductions in body fat percentage and visceral fat rating, together with the preservation of fat-free mass, suggest favorable changes in body composition quality following the intervention. Although fat mass decreased in the intervention group over the study period, the between-group difference in fat mass change was not statistically significant. Therefore, the observed improvements in body composition are primarily reflected by the reduction in body fat percentage while maintaining lean mass. Maintaining lean mass during weight loss is metabolically beneficial, as skeletal muscle mass is closely linked to insulin sensitivity and resting energy expenditure, thereby helping longer-term weight maintenance and metabolic stability. Importantly, this pattern of selective fat loss suggests that the SBB may promote favorable energy partitioning rather than indiscriminate tissue catabolism.

Baseline dietary fibre intake was low in both groups, which may reflect both inadequate habitual consumption of fibre-rich foods and the inherent limitations of self-reported dietary assessment methods. Dietary records are susceptible to reporting inaccuracies and underestimation of food intake, particularly among free-living adults. Nevertheless, a substantial increase in fibre intake was observed following the intervention, indicating good adherence to the prescribed beverage consumption and a meaningful improvement in overall dietary fibre exposure during the intervention period.

One of the key mechanisms underlying these changes appears to be related to dietary modification. The intervention group demonstrated a substantial increase in dietary fibre intake, accompanied by increases in carbohydrate and protein intake. Therefore, the observed improvements in anthropometric outcomes should not be attributed exclusively to dietary fibre intake. Rather, the intervention appears to have altered the overall dietary composition, with dietary fibre likely playing an important contributory role. Dietary fibre has been shown to enhance satiety, delay gastric emptying, and modulate postprandial glycaemic responses, which may help reduce excess energy intake. In addition, fermentation of dietary fibre in the colon produces short-chain fatty acids (SCFAs), such as acetate and propionate, which have been implicated in appetite regulation, lipid oxidation, and improved metabolic flexibility [35,36,37]. Concurrently, increased protein intake may also have contributed to enhanced satiety and the preservation of fat-free mass during weight reduction. Collectively, these dietary changes may explain the preferential reduction in adiposity observed in the present study.

Dietary fibre has been shown to delay gastric emptying, enhance gastrointestinal distension, and stimulate the release of key satiety hormones such as peptide YY (PYY) and glucagon-like peptide-1 (GLP-1), thereby contributing to prolonged appetite regulation. In the present study, satiety responses assessed using VAS indicated a progressive improvement in appetite-related perceptions over time, with the intervention group demonstrating consistent reductions in hunger, desire to eat, and prospective food consumption, particularly by Week 12. Notably, significant group effects were observed for desire to eat snacks and prospective food consumption, suggesting that the intervention may have influenced anticipatory and hedonic aspects of eating behavior rather than immediate satiation.

These findings align with contemporary appetite regulation theory, which distinguishes between homeostatic hunger (physiological need) and hedonic appetite (reward-driven eating). The observed reduction in snack-related desire suggests improved regulation of reward-driven eating, a key contributor to excess energy intake and adiposity. Such modulation of non-homeostatic eating behaviour is particularly relevant in free-living populations, where dietary choices are often influenced by environmental and psychological cues. Consequently, even modest but sustained improvements in appetite control may translate into cumulative reductions in energy intake over time. This provides a plausible explanation for the observed improvements in body weight and adiposity, highlighting the role of dietary fibre not only in physiological satiety mechanisms but also in shaping eating behaviour patterns that support long-term weight management.

Although body composition and adiposity improved significantly, the intervention maintained stable profiles for glucose, lipid parameters, inflammatory markers (CRP and IL-6), as well as renal and liver function indices, indicating that regular consumption of SBB did not disrupt metabolic homeostasis. Besides that, fasting blood glucose improved over time (*p* < 0.001), suggesting a favourable trend in glycaemic regulation. Importantly, although the SBB contains 15.48 g/100 g total sugars, the actual daily intake remained moderate due to controlled twice-daily consumption. The absence of adverse metabolic effects indicates that the overall nutritional matrix, characterised by high dietary fibre content, may have attenuated the glycaemic impact of sugars through delayed gastric emptying and reduced glucose absorption. This highlights the importance of food matrix interactions, where fibre-rich formulations can mitigate potential metabolic risks associated with sugar content. Therefore, these findings show that SBB is metabolically well-tolerated and supports its suitability as a functional dietary strategy for weight and metabolic health management.

In terms of safety, no clinically significant adverse changes were observed in biochemical parameters, including glucose, lipid profile, renal function markers, C-reactive protein, and IL-6, during the intervention period. The lack of significant between-group differences in biochemical outcomes may be attributed to several factors, including the relatively healthy baseline status of participants, short intervention duration, and sample size limitations. It is well established that metabolic biomarkers such as lipid profile and inflammatory markers often require longer intervention periods or higher baseline risk to exhibit significant changes.

However, participant withdrawals due to product-related issues were recorded. This included an inability to tolerate the taste of the intervention product and gastrointestinal symptoms, specifically diarrhea in one participant. Although these events were limited in number and not severe, they indicate that gastrointestinal responses and product acceptability may vary among individuals. These observations suggest that biochemical outcomes alone may not fully reflect the overall safety and acceptability profile of dietary interventions.

The findings of this study contribute to the growing body of evidence supporting the role of functional, fibre-enriched soy-based beverages in weight management. Unlike interventions that rely solely on caloric restriction, the present approach highlights the potential importance of broader dietary modifications achieved through a fibre-enriched soy-based beverage. While increased dietary fibre intake likely contributed to the observed benefits through enhanced satiety and appetite regulation, concurrent increases in protein and carbohydrate intake indicate that the intervention influenced overall dietary composition. Therefore, the improvements in adiposity and body composition are likely the result of multiple interacting dietary mechanisms rather than the effect of dietary fibre alone.

Several limitations should be considered when interpreting the findings of this study. First, although cluster randomization was employed, only five clusters were included, which may have limited statistical power at the cluster level and reduced the ability to account for cluster-level variation adequately. Furthermore, the statistical analyses were conducted at the individual-participant level and did not explicitly account for clustering effects. As a result, the variance of treatment estimates may have been underestimated, potentially increasing the likelihood of type I error. Therefore, the findings should be interpreted with caution, and future studies should include a larger number of clusters and statistical approaches that appropriately account for cluster-level correlation.

Second, the study utilized an open-label design without a placebo comparator, which may have introduced performance and expectation bias. Participants in the intervention group received the dietary fibre beverage together with compliance monitoring and regular follow-up, whereas the control group continued their habitual lifestyle without a comparable placebo intervention. Consequently, subjective outcomes, particularly appetite and satiety assessments, may have been influenced by participants’ awareness of treatment allocation. Although the primary outcomes, including anthropometric, body composition, and biochemical measures, were objectively assessed using standardized procedures and are therefore less susceptible to such bias, future studies should consider placebo-controlled, double-blind designs with equivalent participant contact between groups. Third, dietary intake was assessed using self-reported dietary records, which are inherently subject to recall error, reporting bias, and underreporting. The relatively low baseline dietary fibre intake observed in both groups may therefore reflect both inadequate habitual fibre consumption and limitations associated with self-reported dietary assessment methods. Accordingly, dietary intake findings should be interpreted with caution. Fourth, while the intervention substantially increased dietary fibre intake, concurrent changes in other dietary components, including protein and carbohydrate intake, were also observed. Therefore, the independent contribution of dietary fibre to the observed improvements in anthropometric and body composition outcomes cannot be definitively established. The beneficial effects observed may reflect the combined influence of increased dietary fibre intake together with broader dietary modifications. Fifth, six participants were excluded from the final analyses because of withdrawal or protocol violations, and a formal intention-to-treat analysis was not performed. Consequently, the results were based on participants with available follow-up data, which may have introduced attrition bias and potentially affected the robustness of the findings. Future studies should incorporate intention-to-treat analyses and appropriate methods for handling missing data to strengthen the validity of the conclusions.

Finally, although the primary outcomes were pre-specified and focused on anthropometric and body composition measures, the study evaluated a broad range of secondary outcomes, including biochemical, inflammatory, dietary intake, and appetite-related parameters. The assessment of multiple secondary endpoints increases the possibility of type I error, whereby some statistically significant findings may have occurred by chance. Therefore, findings from secondary and exploratory analyses should be interpreted cautiously and warrant confirmation in larger studies with pre-specified outcome hierarchies and appropriate approaches to address multiplicity.

## 5. Conclusions

In conclusion, the SBB intervention was associated with improvements in body weight, central adiposity, and body fat percentage over the 12-week study period. These effects may be related to sustained dietary composition changes and appetite-related behavioral modulation rather than acute satiety responses. No clinically significant adverse changes were observed in the measured biochemical parameters, including glucose, lipid profile, renal function markers, C-reactive protein, and IL-6, suggesting short-term tolerability of the intervention. However, participant withdrawals related to product acceptability and gastrointestinal symptoms, particularly diarrhea, indicate that individual responses may vary and that biochemical outcomes alone may not fully reflect the overall tolerability and acceptability profile of dietary interventions. Therefore, these findings suggest the potential efficacy of fibre-based ready-to-consume beverages as a practical approach to increasing dietary fibre intake and improving adiposity-related outcomes in free-living populations. Further studies with larger sample sizes, longer intervention durations, and systematic assessment of adverse events and product acceptability are warranted to confirm both efficacy and longer-term safety.

## Figures and Tables

**Table 1:** Baseline characteristics for all subjects.

	Control Group (*n* = 21)	Intervention Group (*n* = 21)	*p*-Value
Age (years), mean (±SD)	35.71 ± 5.96	38.43 ± 8.05	0.222
**Anthropometric parameters, mean (±SD)**			
Height (cm)	157.48 ± 8.72	159.95 ± 5.93	0.226
Weight (kg)	66.69 ± 7.90	70.29 ± 7.52	0.138
BMI (kg/m^2^)	26.89 ± 2.15	27.41 ± 1.77	0.407
WC (cm)	82.20 ± 8.10	83.59 ± 8.06	0.580
HC (cm)	101.19 ± 5.36	100.60 ± 4.80	0.441
WHR	0.81 ± 0.07	0.83 ± 0.08	0.384
**Blood pressure, mean (±SD)**			
Systolic BP (mmHg)	123.71 ± 13.53	121.90 ± 13.21	0.687
Diastolic BP (mmHg)	81.90 ± 9.81	81.43 ± 9.59	0.874
**Body composition, mean (±SD)**			
BF%	35.37 ± 6.26	35.40 ± 5.83	0.930
Fasting Plasma Glucose (mmol/L)	5.16 ± 0.47	5.03 ± 0.37	0.330
HbA1c (%)	5.68 ± 0.33	5.64 ± 0.27	0.453
Urea (mmol/L)	4.03 ± 1.19	4.38 ± 0.98	0.241
Creatinine (µmol/L)	70.90 ± 14.73	69.14 ± 11.98	0.850
eGFR (mL/min)	102.6 ± 25.08	110.19 ± 15.79	0.521
Total cholesterol (mmol/L)	4.98 ± 1.24	5.56 ± 1.24	0.137
LDL cholesterol (mmol/L)	3.10 ± 0.95	3.62 ± 1.19	0.125
HDL cholesterol (mmol/L)	1.46 ± 0.61	1.44 ± 0.25	0.489
Triglycerides (mmol/L)	1.38 ± 0.81	1.14 ± 0.69	0.207
c/HDL	4.00 ± 1.00	4.00 ± 1.23	0.989
Non-HDL (mmol/L)	3.79 ± 0.96	4.12 ± 1.31	0.353
Total protein (g/L)	74.67 ± 3.21	75.71 ± 4.29	0.376
Albumin (g/L)	46.38 ± 2.87	47.52 ± 2.62	0.185
Total bilirubin (µmol/L)	10.02 ± 5.98	10.10 ± 4.97	0.970
Alkaline phosphatase (U/L)	66.85 ± 18.83	68.14 ± 16.83	0.817
AST (U/L)	25.95 ± 15.69	26.33 ± 9.07	0.207
ALT (U/L)	23.48 ± 20.73	27.71 ± 19.97	0.137
C-reactive protein (mg/L)	7.13 ± 14.07	6.93 ± 15.05	0.742
IL-6 (pg/mL)	36.95 ± 34.15	36.81 ± 37.32	0.806

BMI—Body Mass Index; WC—Waist Circumference; HC—Hip Circumference; WHR—Waist–Hip ratio; BP—Blood Pressure; BF%—Body Fat Percentage; eGFR—estimated Glomerular Filtration Rate; HDL—High Density Lipoprotein; LDL—Low Density Lipoprotein; c/HDL—Cholesterol to HDL Cholesterol Ratio; AST—Aspartate Aminotransferase; ALT—Alanine Aminotransferase; IL-6—Interleukin-6; SD—Standard Deviation.

**Table 2:** Changes in anthropometric parameters.

Measures	Mean (±SD)										*p*-Value		
	Week 0		Week 4		Week 8		Week 12		Change (Week 12–Week 0)		Group ^a^	Time ^a^	Group × Time ^a^
	CG (*n* = 18)	IG (*n* = 15)	CG (*n* = 18)	IG (*n* = 15)	CG (*n* = 18)	IG (*n* = 15)	CG (*n* = 18)	IG (*n* = 15)	CG (*n* = 18)	IG (*n* = 15)			
Weight (kg)	67.02 ± 8.24	69.70 ± 8.68	66.93 ± 8.40	69.85 ± 8.89	67.36 ± 8.59	69.13 ± 8.75	67.51 ± 8.36	68.58 ± 9.04	0.49 ± 0.80	−1.12 ± 1.66 *^b^	0.486	0.372	<0.001 *
BMI (kg/m^2^)	26.67 ± 2.16	27.23 ± 1.86	26.67 ± 2.24	27.29 ± 1.91	26.77 ± 2.39	27.03 ± 1.82	26.84 ± 2.18	26.80 ± 1.93	0.18 ± 0.37	−0.43 ± 0.65 *^b^	0.625	0.507	0.021 *
WC (cm)	81.96 ± 8.56	84.43 ± 9.15	84.64 ± 9.34	82.51 ± 9.50	81.44 ± 7.75	81.13 ± 8.03	80.17 ± 8.87	80.13 ± 8.94	−1.79 ± 2.43	−4.29 ± 3.46 *^b^	1.000	0.024 *	0.046 *
HC (cm)	100.61 ± 5.32	99.70 ± 5.42	99.86 ± 6.19	98.60 ± 6.48	98.42 ± 4.13	97.13 ± 5.30	98.67 ± 4.30	96.73 ± 6.20	−1.94 ± 2.85	−2.97 ± 4.70	0.456	<0.001 *	0.818
WHR	0.81 ± 0.07	0.85 ± 0.08	0.85 ± 0.07	0.84 ± 0.07	0.83 ± 0.08	0.83 ± 0.06	0.81 ± 0.08	0.83 ± 0.06	−0.002 ± 0.03	−0.02 ± 0.05	0.645	0.046 *	0.042 *
**Blood Pressure**													
Systolic BP (mmHg)	124.11 ± 13.69	122.13 ± 14.59	120.22 ± 14.19	121.87 ± 8.03	122.89 ± 13.23	122.73 ± 9.44	120.83 ± 12.19	118.40 ± 9.04	−3.28 ± 10.33	−3.73 ± 9.82	0.827	0.172	0.796
Diastolic BP (mmHg)	83.33 ± 9.20	81.20 ± 10.83	78.11 ± 8.05	82.27 ± 6.69	78.72 ± 9.25	80.60 ± 7.38	78.11 ± 8.30	81.00 ± 6.58	−2.33 ± 7.15	−0.20 ± 8.26	0.419	0.459	0.217

CG—Control Group; IG—Intervention Group; BMI—Body Mass Index; WC—Waist Circumference; HC—Hip Circumference; WHR—Waist–Hip ratio; BP—Blood Pressure; SD—Standard Deviation. * Significance at the *p* < 0.05 level. ^a^ Linear mixed-effects model was applied. ^b^ Independent *t*-Test was applied.

**Table 3:** Changes in body composition.

Measures	Mean (±SD)										*p*-Value		
	Week 0		Week 4		Week 8		Week 12		Change (Week 12–Week 0)		Group ^a^	Time ^a^	Group × Time ^a^
	CG (*n* = 18)	IG (*n* = 15)	CG (*n* = 18)	IG (*n* = 15)	CG (*n* = 18)	IG (*n* = 15)	CG (*n* = 18)	IG (*n* = 15)	CG (*n* = 18)	IG (*n* = 15)			
BF%	34.58 ± 6.40	34.00 ± 6.26	35.07 ± 7.29	33.75 ± 6.45	35.11 ± 6.68	33.65 ± 6.04	34.93 ± 6.31	33.27 ± 6.32	0.35 ± 0.67	−0.73 ± 0.89 *^b^	0.580	0.296	0.004 *
Fat mass (kg)	23.23 ± 4.55	23.60 ± 4.85	23.53 ± 5.01	23.46 ± 4.84	23.73 ± 5.03	23.15 ± 4.59	23.61 ± 4.59	22.70 ± 4.77	0.38 ± 0.72	−0.9 ± 1.10 *^b^	0.856	0.503	0.305
Fat-free mass (kg)	44.01 ± 8.58	46.10 ± 8.08	43.36 ± 9.19	46.41 ± 8.49	43.96 ± 8.49	45.98 ± 8.08	43.31 ± 8.04	46.69 ± 8.11	0.59 ± 11.18	−0.7 ± 12.26	0.266	0.834	0.047 *
Visceral fat rating	8.06 ± 2.41	9.40 ± 3.04	8.06 ± 2.41	9.47 ± 3.16	8.11 ± 2.45	9.13 ± 3.13	8.06 ± 2.41	9.00 ± 3.21	0.00 ± 0.34	−0.40 ± 0.63	0.230	0.011 *	0.005 *
Muscle mass (kg)	41.59 ± 8.23	43.51 ± 7.78	39.58 ± 10.90	43.80 ± 8.17	39.66 ± 10.38	43.39 ± 7.77	41.62 ± 8.11	43.32 ± 8.02	0.02 ± 0.44	−0.19 ± 0.75	0.338	0.427	0.270

CG—Control Group; IG—Intervention Group; BF%—Body Fat Percentage. * Significance at the *p* < 0.05 level. ^a^ Linear mixed-effects model was applied. ^b^ Independent *t*-Test was applied.

**Table 4:** Changes in biochemical parameters.

Measures	Mean ± SD						*p*-Value		
	Week 0		Week 12		Change (Week 12–Week 0)		Group ^a^	Time ^a^	Group × Time ^a^
	CG (*n* = 18)	IG (*n* = 15)	CG (*n* = 18)	IG (*n* = 15)	CG (*n* = 18)	IG (*n* = 15)			
Fasting blood glucose (mmol/L)	5.15 ± 0.41	5.05 ± 0.39	4.86 ± 0.35	4.75 ± 0.58	−0.29 ± 0.27	−0.30 ± 0.35	0.278	<0.001 *	0.640
HbA1c (%)	5.63 ± 0.32	5.64 ± 0.31	5.60 ± 0.30	5.57 ± 0.50	−0.03 ± 0.13	−0.07 ± 0.36	0.741	0.373	0.592
Total cholesterol (mmol/L)	4.97 ± 1.03	5.73 ± 1.33	5.10 ± 0.67	5.50 ± 1.17	0.13 ± 1.07	−0.23 ± 0.93	0.313	0.847	0.260
LDL cholesterol (mmol/L)	3.12 ± 0.80	3.79 ± 1.27	3.22 ± 0.66	3.58 ± 1.08	0.09 ± 0.76	−0.21 ± 0.97	0.297	0.863	0.243
HDL cholesterol (mmol/L)	1.51 ± 0.67	1.42 ± 0.28	1.33 ± 0.24	1.35 ± 0.24	−0.18 ± 0.49	−0.07 ± 0.14	0.826	0.082	0.507
Triglycerides (mmol/L)	1.27 ± 0.67	1.19 ± 0.81	1.19 ± 0.65	1.27 ± 0.65	−0.08 ± 0.71	0.08 ± 0.51	0.836	0.915	0.545
c/HDL-C	3.96 ± 0.74	4.19 ± 1.34	3.90 ± 0.71	4.26 ± 1.31	−0.06 ± 0.76	0.07 ± 0.73	0.725	0.949	0.564
Non-HDL cholesterol (mmol/L)	3.79 ± 0.54	4.31 ± 1.40	3.78 ± 0.64	4.16 ± 1.25	−0.01 ± 0.50	−0.15 ± 0.96	0.505	0.650	0.507
Urea (mmol/L)	3.98 ± 1.00	4.51 ± 1.02	3.87 ± 1.07	4.41 ± 1.45	−0.11 ± 1.09	−0.10 ± 0.75	0.235	0.805	0.844
Creatinine (µmol/L)	70.06 ± 13.57	71.20 ± 13.61	69.72 ± 15.76	68.80 ± 11.47	−0.34 ± 6.61	−2.40 ± 7.46	0.715	0.792	0.658
eGFR (mL/min)	107.72 ± 10.59	108.33 ± 15.85	107.22 ± 13.98	109.53 ± 13.99	−0.50 ± 8.48	1.2 ± 8.54	0.365	0.290	0.401
Total protein (g/L)	75.00 ± 3.29	75.40 ± 3.50	75.61 ± 2.91	75.00 ± 4.38	0.61 ± 3.73	−0.40 ± 4.66	0.559	0.428	0.921
Albumin (g/L)	47.00 ± 2.50	47.40 ± 2.92	47.22 ± 2.01	48.27 ± 2.91	0.22 ± 2.13	0.87 ± 2.77	0.894	0.032 *	0.078
Total bilirubin (µmol/L)	10.36 ± 6.39	10.53 ± 5.48	10.57 ± 6.86	12.00 ± 4.97	0.19 ± 5.64	1.47 ± 3.25	0.278	0.357	0.620
Alkaline phosphatase (U/L)	68.17 ± 19.78	69.53 ± 18.45	69.33 ± 12.86	72.07 ± 20.22	1.17 ± 15.92	2.53 ± 6.80	0.634	0.804	0.939
AST (U/L)	25.89 ± 16.50	29.40 ± 8.93	24.00 ± 10.15	26.47 ± 9.98	−1.89 ± 18.02	−2.93 ± 5.00	0.108	0.347	0.093
ALT (U/L)	23.17 ± 21.47	33.60 ± 20.74	28.44 ± 32.96	35.60 ± 24.47	5.28 ± 13.37	2.00 ± 10.55	0.359	0.987	0.218
GGT (IU/L)	27.06 ± 18.68	42.27 ± 33.12	26.94 ± 20.05	43.07 ± 35.69	−0.11 ± 4.51	0.80 ± 22.43	0.050 *	0.022 *	0.026 *
C-reactive protein (mg/L)	7.56 ± 14.78	7.74 ± 16.39	4.54 ± 5.27	3.30 ± 2.58	−3.02 ± 13.64	−4.44 ± 14.81	0.601	0.647	0.707
IL-6 (pg/mL)	38.58 ± 35.03	37.61 ± 38.59	40.47 ± 33.58	37.11 ± 30.28	1.89 ± 42.76	−0.50 ± 46.37	0.664	0.090	0.936

CG—Control Group; IG—Intervention Group; HbA1c—hemoglobin A1c; LDL—Low-Density Lipoprotein; HDL—High-Density Lipoprotein; c/HDL-C—Cholesterol to HDL Cholesterol Ratio; eGFR—estimated Glomerular Filtration Rate; AST—Aspartate Aminotransferase; ALT—Alanine Aminotransferase; GGT—Gamma-Glutamyl Transferase; IL-6—Interleukin-6; SD—Standard Deviation. * Significance at the *p* < 0.05 level. ^a^ Linear mixed-effects model was applied.

**Table 5:** Changes in diet.

Measures	Week 0		Week 4		Week 8		Week 12	
	CG (*n* = 18)	IG (*n* = 15)	CG (*n* = 18)	IG (*n* = 15)	CG (*n* = 18)	IG (*n* = 15)	CG (*n* = 18)	IG (*n* = 15)
Energy (kcal/day)	1351.05 ± 379.72	1546.18 ± 557.64	1489.32 ± 763.81	1548.28 ± 356.99	1519.44 ± 564.30	1504.57 ± 532.42	1711.35 ± 591.86	1775.59 ± 546.62
Carbohydrates (g/day)	146.17 ± 40.76	181.03 ± 71.61	182.75 ± 92.28	241.55 ± 61.29 *	184.17 ± 89.78	247.77 ± 73.21 *	201.61 ± 76.61	276.07 ± 69.30 *
Protein (g/day)	62.56 ± 18.13	65.18 ± 22.42	57.32 ± 22.98	81.31 ± 21.98 *	66.54 ± 25.25	94.31 ± 42.14 *	65.42 ± 23.87	98.00 ± 39.85 *
Fat (g/day)	55.13 ± 20.15	58.87 ± 21.84	57.65 ± 35.67	64.09 ± 13.30 *	56.08 ± 22.19	65.00 ± 21.25	67.12 ± 24.24	74.04 ± 21.14
Dietary fibre (g/day)	3.04 ± 1.53	3.09 ± 2.60	5.21 ± 4.68	48.14 ± 9.64 *	4.98 ± 4.90	50.15 ± 6.65 *	5.94 ± 4.34	50.42 ± 5.17 *

CG—Control Group; IG—Intervention Group. * Significance between groups at the *p* < 0.05 level based on time.

**Table 6:** Mean daily changes in subjective appetite ratings (ΔVAS) averaged across meals at each study timepoint.

	ΔVAS (Mean ± SEM)						*p*-Value		
	Week 0		Week 4		Week 12		Group	Time	Group × Time
	CG (*n* = 18)	IG (*n* = 15)	CG (*n* = 18)	IG (*n* = 15)	CG (*n* = 18)	IG (*n* = 15)			
Fullness	30.00 ± 4.13	31.71 ± 4.03	32.22 ± 3.51	31.33 ± 3.85	32.04 ± 3.51	36.67 ± 3.85	0.561	0.637	0.756
Hunger	−25.90 ± 4.71	−21.71 ± 4.59	−33.15 ± 4.00	−27.56 ± 4.38	−31.48 ± 4.00	−32.67 ± 4.38	0.421	0.153	0.699
Desire to eat meal	−20.513 ± 4.57	−20.98 ± 4.57	−23.52 ± 3.98	−25.56 ± 4.34	−25.37 ± 3.98	−33.79 ± 4.36	0.305	0.130	0.620
Desire to eat snack	−5.39 ± 3.82	−11.46 ± 3.72	−2.22 ± 3.24	−15.78 ± 3.56	−9.26 ± 3.24	−21.33 ± 3.56	<0.001 *	0.092	0.554
Thirst	−3.33 ± 4.33	−13.17 ± 4.23	−7.69 ± 3.75	−10.67 ± 4.03	−7.69 ± 3.75	−17.78 ± 4.03	0.019 *	0.518	0.578
Prospective food consumption	−14.62 ± 4.42	−21.46 ± 4.31	−18.46 ± 3.83	−25.33 ± 4.11	−16.67 ± 3.76	−30.44 ± 4.11	0.007 *	0.405	0.610

CG—Control Group; IG—Intervention Group; ΔVAS—Changes in subjective appetite sensations; SEM—Standard Error of the Mean. Values are presented as mean ± SEM. ΔVAS was calculated as the difference between post- and pre-meal scores. Differences were analyzed using linear mixed models. * Significance at the *p* < 0.05 level.

## Data Availability

The data that support the findings of this study are available from the corresponding author upon reasonable request.

## Abbreviations

The following abbreviations are used in this manuscript:

ALT	Alanine aminotransferase
AST	Aspartate aminotransferase
BMI	Body Mass Index
BF%	Body fat percentage
BP	Blood pressure
CG	Control Group
CRP	C-reactive protein
c/HDL	Cholesterol to HDL cholesterol
eGFR	estimated Glomerular Filtration Rate
ELISA	Enzyme-linked immunosorbent assay
FFM	Fat-free mass
GGT	Gamma-glutamyl transferase
HbA1c	hemoglobin A1c
HC	Hip circumference
HDL	High-density lipoprotein
IG	Intervention Group
IL-6	Interleukin-6
LDL	Low-density lipoprotein
NRS	Numerical rating scale
SBB	Soy-based dietary fibre
SD	Standard deviation
VAS	Visual analogue scale
WC	Waist circumference
WHR	Waist–Hip ratio
